# Reasons for formula feeding among rural Bangladeshi mothers: A qualitative exploration

**DOI:** 10.1371/journal.pone.0211761

**Published:** 2019-02-26

**Authors:** Atiya Rahman, Fahmida Akter

**Affiliations:** Research and Evaluation Division (RED), BRAC, Mohakhali, Dhaka, Bangladesh; International Telematic University Uninettuno, ITALY

## Abstract

In Bangladesh the exclusive breastfeeding rate remains low and prelacteal, formula and bottle feeding is increasing. This study aims to explore reasons behind infant formula feeding practices from mothers, caregivers, and health care provider’s perspective. This was a qualitative study carried out in four sub-districts of Sylhet and Jessore of rural Bangladesh. Data were collected through focus group discussions (12), in-depth interviews (4) and key informant interviews (12) from January to February 2014. The qualitative data collected and were analyzed using thematic content analysis. This study clearly showed the factor behind formula feeding by Bangladeshi rural women. One of the major findings was that women could not differentiate between formula and other milk. Main differences between formula and powder milk were the type of consumer where formula only was taken by infant and children less than 2 years. Other major reasons include; poor breastfeeding practices, lack of appropriate breastfeeding practices, superficial knowledge on harmful effect on infant formula; perceived insufficient breast milk production, the influence of family and society and authoritarian power of hospital staff. Rural mothers have intension to feed infant formula to their infants due to various factors including individual, social, cultural and institutional. These identified factors can contribute to policy making and develop more specific interventions targeting expected mother and their family members that can contribute to stop formula feeding and increase breastfeeding practices in rural Bangladesh.

## Introduction

Infant feeding practices have a significant effect on childrens’ nutritional state [1]. Early initiation of breast milk substitutes (BMS) and late introduction of semi-solid complementary foods are common practices responsible for the rapid increase in the prevalence of undernutrition among 6–24 months aged children [2]. To achieve optimal growth, development and health of infants, the World Health Organization (WHO) recommends exclusive breastfeeding till 6 months of age [3]. The WHO also recommends that all neonates be breastfed within one hour of birth [4] as early initiation of breastfeeding is associated with protection against diarrhoea and increases the likelihood of longer duration of exclusive breastfeeding which helps with pregnancy- spacing [5]. However, globally only 40% of mothers exclusively breastfeed their children for six months [6]. Delay and poor initiation of breastfeeding infants and young children expose them to various malnutrition and infectious diseases [7]. It is thus well documented that implications on infant health are mostly dependent on whether women breastfeed, for how long and how exclusively [8]. Infants who were exclusively breastfed during the first hour of life were nine times less likely to die than those who were initiated to mixed formula and breast milk within 72 hours of birth [9]. According to a report of WHO, breastfeeding could prevent over three-fourths of deaths in early infancy and 37% of deaths during the second year of life among children [3]. Studies revealed that the initiation of breast milk within 1 hour reduced deaths by 19–22% [7, 10]. Breastfeeding significantly reduces the risk of death especially from diarrhoea in infants compared to formula-fed babies [11]. Despite the enormous benefits of breastfeeding for children and mothers, [3] globally progress on this issue is both uneven and suboptimum [12].

Several studies in developing countries have shown mothers decision on feeding practices [13–15] but those are not well documented in South Asian perspective. A variety of factors have been identified to explain the infant feeding decisions including knowledge, attitude, beliefs as well as socio-cultural and physiological factors [16]. Lack of accessibility on breastfeeding information, cultural relevance and sensitivity of breastfeeding messages have also been identified as influencing infant feeding decisions [17]. Based on some others previous studies on formula feeding in several parts of the world, social embarrassment, commercial pressure, lack of social support, beliefs of family and society, perceived insufficient milk supply and knowledge of infant feeding, maternity leave and mothers/caregivers concerns on infant weight gain are common factors associated with the initiation and continuation of formula feeding [13–15].

There has been limited research in Bangladesh that focuses on the better understanding of why some mothers in recent times choose infant formula to feed their infants. One of such studies suggests that socio-economic factors are associated with the choice to select the appropriate formula feed [18]. This urban-based study clearly showed that the use of breast milk substitute was higher among the mothers of middle socio-economic status (SES) than mothers of low SES. This is due to the fact that these middle SES groups of mothers perceived infant formula as being the best food for their children.

Bangladesh is a lower middle income country with a population of 158 million. Approximately 70% of the population lives in rural areas [19]. According to the Bangladesh Demography and Health Services (BDHS) 2014, the proportion of exclusive breastfeeding was 55% while the median duration of breastfeeding is nine months. The likelihood of receiving prelacteal feed is higher for births assisted by a health professional and delivered at a health facility. The study also showed that six percent of breastfeeding children under 2 months of age are given infant formula. Also in recent years, the rate of bottle feeding has increased in Bangladesh. However, BDHS could not show any precise reasons behind lower rate of exclusive breastfeeding or initiation of infant formula at the early stage of life. This may be due to poor knowledge and awareness of the dangers of bottle feeding among mothers and family members. Ensuring exclusive breastfeeding till 24 months of a baby is still a challenge in our country.

BRAC (formerly known as Bangladesh Rural Advancement Committee) is a non-government organization which was developed as a rehabilitation organization in 1972 in a remote and hard-to-reach area of Bangladesh. Officially BRAC’s mission is to empower people and communities in situations of poverty, illiteracy, diseases and social injustice [20]. From its inception, BRAC has been built on around two overarching goals namely elimination of poverty and empowering the marginalized people, especially women. To achieve these goals BRAC has a diversified programme portfolio for the rural poor with mostly its low-income households offering services such as microfinance, targeting the ultra poor (TUP), agriculture and food security, education, health and nutrition, water, sanitation and hygiene, disaster, environment and climate change, community empowerment, Human rights and legal Aid services, integrated development programme[20].

To achieve the success on millennium development goal (MDG) 4 (Reduce child mortality) and 5 (Improve maternal health by 2015), the BRAC Health, Nutrition and Population Programme (HNPP) was involved in implementing the Alive and Thrive initiatives that focus on reducing malnutrition among under-two children through proper ‘Infant and Young Children Feeding’ (IYCF) practices until 24 months.

BRAC Advocacy for Social Change (ASC) is a programme that works to create an enabling environment for other BRAC programmes so that they can achieve their objectives more successfully. In the case of providing support BRAC ASC has initiated a project, ‘Promote breast milk substitutes code implementation (PBCI)’ for the promotion of breastfeeding practices through increasing awareness of stakeholders on BMS code in Jessore and Sylhet districts facilitating BRAC HNPP [21]. As a part of this project, ASC has been developing a consensus of the various stakeholders from all level such as to aware on the new law of breast milk substitute namely BMS code 2013 through regular roundtables, dialogues, seminar and workshops. Before implementing the project, it was very much appropriate to gather some imperative knowledge about community perception and practice on the issue. So that the findings will be helpful to develop effective strategies for the PBCI project to mobilize and motivate stakeholders who can influence mothers behaviour in-terms of infant feeding practices.

To the best of our knowledge, no research has been undertaken on the reasons of formula feeding practices among rural women in Bangladesh. Therefore this study sets out to explore the reasons why and how some mothers make their decisions to feed formula to their infants.

## Materials and methods

### Ethics statement

The study followed institutional review process of Research and Evaluation Division (RED), BRAC. BRAC Advocacy from Social Change and HNPP programme provided official permission to do the study as no identifying information was collected on study participants. The study was conducted after getting official permission from BRAC Advocacy from Social Change unit. Prior to the interview each respondent was verbally informed about objective of the study from written consent form. They were informed that their participation and signing in written consent form is voluntary and that confidentiality would be secure throughout the research process. Finally interview was done when respondents provided their verbal consent to the study group.

### Study design and site

This is a qualitative study with exploratory in nature. This study was conducted in purposively selected four sub-districts of rural Bangladesh. Two in the north-eastern district of Sylhet in Sylhet division and remaining two in the south western district of Jessore under broader Khulna division. These two districts have been selected purposively as BRAC ASC has initiated a project, “Promote breast milk substitute’s code implementation (PBCI)” for promoting proper IYCF practices through increased awareness of stakeholders on BMS code.

Sylhet is bounded by the Meghalaya state of India on the north, Maulvibazar district on the south, the Assam state of India on the east, Sunamganj and Habiganj districts on the west [22]. On the other hand, Jessore is bounded by Jhenaidah and Magura districts on the north, Satkhira and Khulna districts on the south, Narail and Khulna districts on the east, the West Bengal state of India on the west [22]. As per the Bangladesh Bureau of Statistics 39% and 24% population in Jessore and Sylhet districts respectively poor those belong to the upper poverty line, of which 18% and 20% population are extremely poor (belongs to lower poverty line) [23].

Beside economic status and geographical position, these two districts are different in many health indicators. Infant and under-5 mortality rates are lowest in Khulna division (33 and 41), while the figures for Sylhet are nearly 2 times higher (59 and 80) than that of Khulna division and also well above the national average [24]. Also, there is a regional variation exist in maternal and child health situation in two districts. According to the Bangladesh Health and Demographic Survey 2014, Sylhet has the highest rates of childhood mortality whereas Khulna has the second highest. In case of IYCF practices, it has been seen that regional rate of exclusive breastfeeding is little higher in Sylhet division (52.2%) compared to Khulna (50.5%) [19]. According to BDHS 2014 prelacteal feeding is more common in Khulna (33%) than in Sylhet (18%) [19]. According to MDG progress report 2015, the underweight rate is the highest in Sylhet Division (39.8%) and lowest in Khulna Division (25.5%). It is therefore also reported that stunting is also most prevalent in Sylhet (50%) and lowest in Khulna (28%) [25].

### Study participants

The study population was categorized into four groups: mother, father, caregiver and health provider. Study participants details are provided below in Table 1.

**Table 1:** Types of interviews by respondent categories.

Group	Type of Respondents	Characteristics	Interview type	Number of Interviews
1	Mother	Children who had breastfeed	FGD*	4
	Mother	Children who had formula feed (with/without breastfeed)	IDI**	4
2	Father	Fathers who have children under 2 years.	FGD	4
3	Caregiver	Grandmother, both maternal and paternal aunty, sister and cousin sister	FGD	4
4	Health provider	Formal- Doctor (Pediatrician/Obstetrician/general Physician); nurse;	KII***	12

* Focus group discussion

** In-depth interview

*** Key informant interview

Purposive sampling strategy was applied for sampling criteria and all the study participants were selected according to the specific purpose of the study.

The inclusion criteria for mother were having children less than two years and permanent residents of the study area. They were included as they bear the prime responsibility for infant feeding practices. In this study ‘breastfeeding’ refers to women whose child has received breast milk only and ‘formula feeding’ as women who have formula fed with/without breastfed of their child.

The second and third group of respondents was selected from the same area who have children or grand-children less than two years. It should be noted that these three respondents were not selected from the same household to get a diversified response. We interviewed fathers as they have an important role in Bangladeshi culture as a main decision maker and ability to earn and buy food for the family member. After the mother elderly/young female members played a significant role as a caregiver who takes care of infants and child in the family. For the health provider group, doctors and nurses were selected purposely as key informant (KI) from *Upazila* health complex. *upazila* health complex works at sub-district level, comprising 31 bed with inpatient, outpatient and other supporting services [26].

### Data collection

Data were collected from January to February 2014. Data collection was done by the trained research assistants (RA) who had a social science background and further trained by both authors on objective and content of the research. Semi-structured interview design was used to elicit data from the participants. Four separate interview guides were prepared in Bengali and data collection was done based on these guidelines. Overall we concentrated on five major areas. This includes:

- Knowledge and practice on breastfeeding
- Knowledge and practice on formula feeding
- Barriers (if any) on breastfeeding practices
- Reasons behind formula feeding/Facilitating factors of formula feeding
- Suggestions from participants(if any)

During pretesting the guides it was found that community people are used to term infant formula as powder milk (in local dialect ‘*gura dudh’*) or container milk (in local dialect ‘*koutar dudh*’). That’s why we defined infant formula as ‘a breast-milk substitute formulated industrially in accordance with applicable *Codex Alimentarius* standards, to satisfy the normal nutritional requirements of infants up to between four and six months of age, and adapted to their physiological characteristics [27]. Infant formula may also be prepared at home, in which case it is described as "home-prepared" [28]. Powdered milk was defined as dried milk which is a manufactured dairy product made by evaporating milk to dryness. Participants were asked about the names of the brand when they refer powder milk or container milk for the clarification and distinction between infant formula and dried milk. If they mentioned the brand name of any infant formula then we considered powder/container milk as an infant formula.

The instrument was pretested among a similar group of respondents in similar settings near to Dhaka district. To ensure the validity of the interview a pretest done before the final data collection and literature focusing on decisions that a woman has to make when choosing between breastfeeding and formula feeding [16–18,28]. Data collection has been done based on a saturation process which is defined as when there is enough information to replicate the study and when the ability to obtain additional new information has been attained [29–31].

We did FGDs as one of the main data collection techniques to reveal information about a range of ideas, feelings, consensus, a disagreement that each and every individual within the group has about certain issues [32]. We conducted four FGDs with mother who breastfed their babies during the data collection period [S1 Table], four with father and four with the caregiver of children under 2 years. The FGDs were started with an introduction of the facilitator, moderator and participants and objective of the research and discussion. FGDs were recorded in a written format, as well as on tape so that the correctness of responses could be checked and confirmed afterwards. All the FGDs were conducted in an open place especially yard of a participant’s household. Each FGD consists of 6–7 members. Each FGDs lasted approximately 60 to 120 minutes.

Four in-depth interviews were carried out with the mothers who had practised formula feeding with their children’s. In order to get meaningful in-depth information from rich cases, IDIs were done which also facilitates to get triangulated findings [S2 Table]. In-depth interviews lasted between 45 minutes to an hour and conducted in the respondents’ home separately from other family members.

Total 12 KIIs was performed with the health care providers in the respective study area. They were interviewed informally to get different perspective regarding feeding infant formula by rural mother. Questions in KII broadly related to experiences and understandings of mother and her families decision on infant and child feeding [S3 Table]. KIIs took place in the health centre and lasted between 40–90 minutes. All the healthcare providers in the selected sites were engaged in this study based on their availability and willingness to participate in the study. The KIIs were conducted at the end of the fieldwork to clarify the questions to be used during interviews with mother’s fathers and caregivers. This was done until the interviews reached saturation stage where new information could not be generated. After each interview, we discussed on the information given by the respondents. We did not get any new information from 10th participants. To get confirmation about saturation level we did 2 more interviews with health providers.

### Data processing and analysis

The interviews and notes were taken by the RAs during the field interview under the guidance of both authors. The interviews were recorded on tape by RAs. The field notes were expanded after each interview on the same day and later verbatim transcripts were prepared in Bengali using expanded field notes and recorded interviews. After completion of verbatim transcripts in Bengali, the most relevant quotes were translated into English by FA and checked by AR to ensure agreement of a correct version of a text. As both the authors did not participate in all the interviews, this translation process was useful for acquiring the full essence of each interview and validity of data interpretation. Also, there was a risk to change the meaning of the actual word if the translation done by the RAs. For example, in the translation phase, we did not translate the terminology what exactly used by the respondent for infant formula. For example, some respondents termed infant formula as ‘*Koutar dudh’* (In English equivalent to container milk) and ‘*Gura dudh’* (In English equivalent to milk powder). Therefore in the whole report, we are using local terminology for infant formula.

Data collected from all groups were subject to the rigorous content analysis described by Graneheim [33]. Firstly we read all the transcripts several times to familiarize the dataset and created initial code list. All texts were then assigned basic codes to identify important features. The codes were grouped into common categories. Finally, categories and themes were developed on the basis of the codes. The transcribed data were analyzed individually by us. During the analysis phase, we met several times and participated in developing sub-categories, categories and themes. We did not perform any software-based analysis due to a small number of sample size. Thus data were analyzed manually. Data triangulation was done by comparing findings from in-depth interviews, FGDs, KIIs and informal discussions (S4 Table).

Trustworthiness of the findings was established using the strategies proposed by Shenton[34]. These strategies include credibility, transferability, dependability and conformability. For dealing with credibility we confirmed the data with the participants at the end of each interview session as peer briefing. Prolonged engagement with the participants and member checking has been done to get credible results. Transferability demonstrates that the research was conducted in such a way that the results can be generalized to the population from which the sample was drawn. We took sample size following purposive sampling procedure. Although the sample size was very limited the findings can be generalized with rural communities of Bangladesh. Dependability means the replication of the same study with the same respondents in a similar context. To confirm dependability, certain approaches were taken such as triangulation and data collection techniques. Conformability was established through hearing the interview, reading the transcribed data several times to confirm the relationship between responses, categories and themes with the entire text which was another process of data triangulation.

## Results

### Participant characteristics

Table 2 presents socio-demographic characteristics of the study participants. Mothers were mostly in the 25 to 34 years age group. Most of them did not attend school (13) but the almost similar number of women (14) did not a complete secondary school certificate examination. The age range of fathers was 25–54 years. The educational qualification of both mother and father groups were similar. Only seven out of forty women and ten out of 36 men had completed secondary school certification and above. Caregivers included grandmother/maternal aunt/paternal aunt/ elderly sister and who were in the age range of 15 to 57 years. Nearly half of the respondents in the caregiver category were literate. Majority of the mothers and caregivers were predominantly housewives, while fathers were engaged in various employment categories such as service, agriculture, driver, day labourers, business etc.

**Table 2:** Demographic characteristics of mothers, fathers and caregivers.

Characteristics	Mothers (N = 40)		Fathers (N = 36)		Caregivers (N = 31)	
	n	%	n	%	n	%
**Age**						
- 15–24	15	37.50	-	-	9	29.03
- 25–34	19	76.0	9	25.0	3	9.67
- 35–44	2	5.0	13	36.11	4	12.90
- 45–54	-	-	14	38.88	11	35.48
- ≥55	-	-	-	-	4	12.90
**Education**						
- No schooling	13	32.50	10	27.77	15	48.38
- Primary incomplete	1	2.50	3	8.33	-	-
- Primary complete	1	2.50	1	2.77	5	16.12
- Secondary incomplete	14	35.0	12	33.33	7	22.58
- Secondary complete and higher	7	17.50	10	27.77	5	16.12
**Occupation**						
- Housewife	35	87.0	-	-	29	93.54
- Service	1	2.0	1	2.77	-	-
- Agriculture	-	-	9	25.00	-	-
- Driver	-	-	2	5.55	-	-
- Day labourer	-	-	1	2.77	-	-
- Business	-	-	16	44.44	-	-
- Others	-	-	4	11.11	2	6.45

Twelve healthcare providers were recruited for the study. The doctors (*n* = 8) and nurses (*n* = 4) were key health providers at *Upazila* health complex who provide basic MNCH services at hospital level only.

The analysis identified six major themes that acted as influencing factors behind formula feeding practices of the mother: (a) good knowledge but poor practice of mother on breastfeeding (b) perceived definition and sources of information on infant formula, (c) superficial knowledge on disadvantage of infant formula (d) perception of insufficient breast milk production (e) social and cultural aspects and (f) hospital practice leads to formula feeding.

### Theme 1: Good knowledge but poor practice of mother on breastfeeding

Mother’s knowledge of breastfeeding practices was explored through conducting FGDs and IDI’s. Majority of the mothers appeared to have adequate knowledge about colostrum feeding, initiation of breastfeeding within 1 hour after delivery, exclusive breastfeeding up to 6 months, appropriate complementary feeding and continuation of breastfeeding till 2 years of age. Some of the responses regarding the benefit of breastfeeding and colostrum feeding were:

*“This is nutritious and good for baby*’*s brain and health development”*

“Colostrum is like first immunization for the baby”

“Breast milk is full of vitamins”

*“There are lots of benefits on breastfeeding*. *For example*, *breast milk protects the baby’s health from several diseases*. *For example pneumonia”*

Health care providers also responded in the same manner. A doctor said:

*“In the past*, *most of the mothers did not know about the proper feeding practice of child*, *but now almost all of them are aware of it*.*”*

The benefits of colostrum feeding were commonly perceived by mothers as ‘the first vaccination of child’. Many of them stated that ‘colostrum prevents life-threatening diseases of babies’.

However, it seemed that they were convinced about the importance of colostrum feeding, but in reality, often mothers could not manage to practice it. Most of the mothers knew how long a baby should be exclusively breastfed and heard about exclusive breastfeeding before from television and local community health workers. Exclusive breastfeeding practice did not also commensurate with the mother’s existing knowledge. A number of barriers to exclusive breastfeeding practices were identified from the interview with respondents. The most commonly mentioned reasons by both groups of mothers were their perception of a lack of satiety of baby, sickness, poor nutritional status etc. During the FGD session with breast milk feeders, mothers shared their experiences why they discontinued exclusive breastfeeding.

*Interviewer*: *Do you know how long a baby should be exclusively breastfed*?

*Respondents (all)*: *6 months*.

*Interviewer*: *How did you know this information?*

*Respondents (all)*: *Shebika apa (community health worker) told*, *I have seen about it from TV*.

*Interviewer*: *Please share your experience how many of you exclusively breastfed your baby*? *That means did not feed anything like water*, *cow’s milk*, *other milk*, *sweets or anything*.

*Respondents 3*: *before 6 months I fed breast milk and shuji (semolina)*

*Interviewer*: *Why*?

*Respondent 3*: *Because she was not happy with breastfeeding*. *She had an appetite*.

*Interviewer*: *How did you feel your baby’s appetite*?

*Respondent 3*: *Because she cried after breastfeeding*.

*Interviewer*: *Anyone wants to share your experience on discontinuation of exclusive breastfeeding*?

*Respondent 5*: *My baby has been started complementary food during 3 months of age*. *She was also not happy with breastfeeding because of my insufficient milk*.

*Interviewer*: *How do you know that you have insufficient breast milk*.

*Respondent 5*: *Actually I was sick then*. *I fed semolina too*.

Health care providers also identified some reasons why some rural mothers discontinued exclusive breastfeeding and initiated infant formula feeding before 6 months. They mentioned about a lack of mother’s knowledge on feeding procedure, proper positioning, and attachment of the baby during breastfeeding; sick/malnourished mother, caesarean delivery, mothers misconception about the growth and development of the baby, and mother’s perception of insufficient breast milk. Nurses and doctors were also identified mothers psychological problem related to breastfeeding as another most important reason that hinders the baby to feed breast milk. They reported that mothers are always annoyed with their cracked nipple, patience and feeling of insufficient breast milk and applied tinned milk as an alternative solution: One health provider pointed out the issue of mothers perception on insufficient breastmilk production with breastfeeding procedure and positioning:

*“At present*, *almost all mothers have knowledge about the importance of breastfeeding but they do not know the proper feeding procedure such as positioning and holding technique during breastfeeding*. *Now a day’s mother’s become very much impatient when they find any difficulty in breastfeeding and they look for instant alternative solutions*.*”*

The above-mentioned quotation concentrates the issue on unawareness of the breastfeeding procedure of mother.

### Theme 2: Perceived definition and sources of information on infant formula

We have questioned all mothers, fathers and grandmothers about feeding practices of their child; whether they used any formula and how they defined formula. Their understanding of formula was determined largely by their perceptions of powder milk/tinned milk, as it is available in markets in a tin box in powder form. They were also asked about the differences between other powdered milk and infant formula brands. Majority of the respondents replied that formula is only for the baby. Again they asked how they know about brands. Most of them said that TV and close relatives were the major sources of information regarding the brand's name of formula. The major sources of information on infant formula are present in Table 3.

**Table 3:** Sources of information on infant formula.

Respondent ID	Sources of information					
	Doctors/ Nurses	TV advertisement	Relatives (Mother/grandmother/sister/brother)	Neighbours	Village doctor/Drug seller at pharmacy	Shop keeper
**Breast milk feeder mother**	√	√	√	√	**×**	×
**Formula feeder mother**	√	√	√	×	**×**	×
**Father**	√	√	√	√	**×**	√
**Caregiver**	√	√	**×**	√	**×**	**×**
**Health care provider**	√	√	√	√	√	√

It was found that formula feeding mothers were mostly influenced by the medical professionals, TV advertisement and relatives as well as family members. Other group of participants placed their opinion that along with those three sources neighbours of the mother, shopkeepers and village doctors or drug seller of a pharmacy store has a greater influence on formula feeding decisions. The findings here are very noticeable that medical professionals did not avoid them to mention as one of the influential factors which was further reinforced by the TV advertisement. Surprisingly, no groups of participants mentioned male/husbands as one of the important sources.

### Theme 3: Superficial knowledge on disadvantage of infant formula

Participant’s knowledge about the advantages and disadvantages of formula feeding were also revealed in the study. From interviews and FGDs with mothers, it was found that most of the participants had little or no clear knowledge on advantages of formula feeding as they could not comprehend the short/long term consequences of formula feeding. Study participants knowledge on infant formula are provided in Table 4. It is also clearly found that they have superficial knowledge on disadvantages though they have mentioned some health risks of formula feeding.

**Table 4:** Respondents perception on infant formula.

Respondents	Advantages	Disadvantages
**Breast milk feeder mother**	No response (mostly), brain development, good health	Any type of disease, diarrhoea, Jaundice, liver problem, gastric, fatty belly, cold and cough, vomiting
**Formula feeder mother**	Full stomach, long sleep, No response	Stomach problem, heart problem, cold and cough, no response, harmful for babies health
**Father**	No response	Diarrhoea, cold and cough, vomiting, mouth infection
**Caregiver**	No advantage	Any type of disease, Stomach pain, diarrhoea, gastric, vomiting, harmful to babies health

The entire formula feeding mother group had C-section delivery and started formula feeding from the hospital according to doctor’s suggestion. All of them stated that after delivery, formula feeding was preferred for its convenience matter. These mothers experienced that after having formula babies tended to get more sleep as their stomach was full for a longer period of time.

Though some mothers were able to mention vomiting or stomach problem’ as an outcome of formula feeding they seemed to be unable to perceive it as serious health concern (Table 4). Mothers treated infant formula as tinned milk (in local term ‘*koutar dudh*’) but perceived container milk as having good nutrition value that helps to develop baby’s overall health. Interestingly this belief was also common to the breast milk feeder mother. Typical responses by mothers were,

*“I heard from my neighbours that if I fed container milk to my child*; *it will help in brain development.”*

FGD and IDI participants mentioned diarrhoea, jaundice, liver problem, gastric, stomach problem, and heart disease, cold and cough as major disadvantages of infant formula. It was noted that formula feeding mothers revealed a low number of problems than breast milk feeding mothers. This was highlighted in another mother in FGDs:

*“Koutar dudh (in English equivalent- tinned milk*) *may be harmful to a baby but I do not know in details about its adverse effect in a child; may be diarrhoea or vomiting or stomach problem* …? *But it is not for the container milk*, *this kind of health problem is very common for a child to suffer at early age.”*

It is actually a common perception of a breastfeeding mother who has no idea about the adverse effect of formula feeding. During discussion sessions, she heard less from others and shared her experience in this way. Actually, she wanted to express that most of the children usually suffered from diarrhoea, vomiting, and stomach problems during their initial stages of life. So it cannot be said that these health problems were created from having formula milk.

### Theme 4: Perception of insufficient breast milk production

Formula feeder mothers also had a perception that only breast milk is not sufficient for proper growth and physical and mental development of a child. All of the children of the formula feeder mothers faced health problems after birth. According to them ‘trouble’ was related to poor sucking and inadequate milk production. One mother started formula feeding her baby after the second day of birth because of sucking difficulties. She explained,

*“I have started formula feeding just after the birth*. *S/he could not suck properly*. *I think it was difficult for my baby because of his/her low birth weight*. *He/she looks really very tiny*. *I have tried a lot but she could not*.*”*

Mothers of twins also stated the same,

*“Since 3 months after their birth, they fed breast milk along with formula. After 3 months my milk production was decreased. I realized as my babies were cried. Then I consulted with a doctor and started ‘L’*.*”*

However few nurses encouraged mother on breastfeeding techniques after delivery. Such comments were highlighted in the following.

*“Within one hour as early after delivery baby should be fed colostrum*. *Mothers usually can't hold baby due to their weakness*, *sickness and if they are for the first time mother*. *That’s why we encourage them and instruct them on breastfeeding*. *That’s why we keep the baby to mother’s breast and teach mothers feeding procedure*. *Most of the time some mothers learn the techniques very quickly*, *sometimes not.”*

All healthcare providers also agreed and found that this kind of perception was common to mothers and their family members. They also justified that having poor maternal nutrition also acted as a reason for insufficient milk production. One doctor described the relation between poor maternal nutrition and insufficient milk production

*“We have seen few mothers had very poor health condition during delivery*. *After delivery baby needs breast milk at the same time mothers is malnourished and weak*. *So they cannot feed properly with the required time*. *It is very common that this time mother felt they have insufficient milk production*.*”*

Regarding insufficient milk production, nurses were replied like doctors and they also thought that poor maternal diet was the main reason behind mother’s insufficient milk production. Their comments were

*“Usually when a woman do not eat good food during pregnancy*, *could have insufficient milk production*. *Sometimes mothers come to me and asked about their insufficient milk production*. *Then we give advise them to eat milk*, *egg*, *vegetables etc*. *we also tell them ‘if you eat more*, *the baby will get more milk from you.”*

### Theme 5: Social and cultural aspects

This study identified two major socio-cultural aspects as influential factors that pushed mothers to feed infant formula. These are discussed under this theme.

#### The culture of social prestige

Having a higher social and economic status also played a significant role in increasing formula feeding. Some breast milk feeder mothers considered themselves poor and felt ashamed to not afford formula. It seems from the interaction that if they have enough money definitely they would buy infant formula for their babies. Feeding infant formula was treated as being of in higher social status within the community. From the mother and caregiver group only educated and rich people could buy formula since it is costly. Some breast milk feeder mothers felt that the reason that they breastfed their children was only due to the cost of infant formula. Few of them explained in FGD session,

*“No*, *I never give tinned milk to our child*. *How could we do that*? *We are poor so we do not have lots of money to buy container milk like rich people”*.

*“Those who have enough money can afford to buy this food (formula)*. *As we do not have money we do breastfeeding.”*

Interestingly, the decision on formula feeding also depends on the mother’s personal choice and interest. One such formula feeder mother was asked in spite of knowing the adverse effect why she fed formula to her baby. She mentioned, *“It’s my choice*. *Formula feeding has several bad impacts on health like heart and liver problem*. *But my baby did not like anything except formula*. *I have tried cow’s milk too*. *But I had nothing to do*. *When my child starts crying I can’t control myself* …*”*

#### Influence from close family members

All of the respondents mentioned that mothers were mostly influenced by their neighbours’, relatives and close family members including grandmother, mother, sister or sister in law. They also work as main information sources for seeking infant formula. For mothers from both groups they regularly saw a relative or friend feeding infant formula to their child and heard about their positive experience regarding it; while feeding formula later to their own child. Mothers became convinced that if they saw a healthy baby they thought the baby took formula milk. It seems that a visual presentation immensely affects the decision making of parents.

In addition, the insistence of elderly family members especially grandmothers were considered as for exclusive breastfeeding. In this context one doctor shared his own experience regarding his mother’s attitude towards infant formula:

*“I am a doctor*. *When my baby was born I failed to convince my mother that nothing is required other than breast milk for the first six of my baby*. *My baby was given formula only because of my mother’s influence*.*”*

The above-mentioned quotation declares that grandmother’s/elderly people’s opinions are highly valued and they often encourage formula supplementation.

However, doctors and nurses believed that family members do not have patience if a mother is suffering from illness or any immediate postoperative complications and pressurizes health professional to give advice for infant formula. A doctor described this issue in this way:

*“Around 95% of caesarean deliveries*, *exclusive breastfeeding practice is not ensured as family members and relatives introduced infant formula to the baby when the mother is in the post-operative period*.*”*

This statement shows that family members and relatives have a great influence on mothers and health professionals to introduce infant formula.

### Theme 6: Hospital practice leads to formula feeding

Mothers also became convinced when doctors suggested infant formula to new mothers or their family members or neighbours. They believed it might be for the betterment of the child. A breast milk feeder mother describes the benefits of infant formula:

*“If there are no benefits then why should doctors prescribe it*? *I feed “X (a brand name of infant formula)” according to doctors’ suggestion and of course he suggested it for my baby’s' well being*.*”*

The decision-making process was not only affected by the visual representation but also having faith in doctors and nurses influenced mothers to suggest infant formula to their child. Such comments were highlighted in the following:

*“As my child always cried; I cannot convince myself that my baby is sufficiently fed so I decided to feed powder milk to my child as I saw my sister-in-law also gives to her child in such situation according to doctors suggestions*.*”*

It can be seen from this quote that this mother was indirectly motivated by her sister in law. She remembered the event that doctors have suggested a formula for her niece/nephew in such circumstances. So she actually convinced herself that this is the right suggestion which pushes her to make such a decision.

However, one formula feeder mother perceived that nurses are the main perpetrator than doctors. Family members of newborn aware that nurses are available to recommend formula when newborn could not suck within one hour. They strongly recommend formula and influenced family members to conceal this issue. A formula feeder mother narrated her situation in this way:

*“When I was in the hospital during delivery a nurse advised my mother in low to give formula to my baby*. *She also threatened us to conceal the issue and said ‘do not disclose to doctors that you are giving formula to your baby*. *It will create a problem between the doctor and me if he/she (doctor) can identify that you are feeding formula as per my advice*. *Also*, *he/she (doctor) kick outs you from this hospital.”*

However, doctors consider this situation differently. As mothers were very much conscious and mentally weak on their baby’s health so it was difficult to convince them otherwise about the negative side of infant formula. One doctor working at district hospital tried to describe the situation:

*“Mothers are very much conscious about their baby’s health and nutrition*. *So*, *the first step is to convince mothers that breast milk is enough for proper growth and development for the first six months of her baby*. *Otherwise*, *for their child’s well-being*, *they will go for formula attracts by advertisement*.*”*

Interestingly doctors and TV advertisements emerged to be the most common sources of information mentioned by mothers too.

Findings of an in-depth interview with formula feeder mother also showed that nurses had an influence on mothers and her family members during as after as delivery to provide infant formula. A formula feeder mother who had c-section delivery and delivery complication said her experience of initiation of infant formula to her baby:

*“After delivery (C-section) I was sick and that time my mother-in-law bought powder milk as per the nurse’s suggestion*. *Since then my baby denies taking breastmilk*. *I tried repeatedly but the baby rejected breast milk though he could easily be fed powder milk using a feeder*.*”*

## Discussion

This study has identified a complex interaction of personal, social and economic factors influences a mother’s decision to breastfeed or breast milk substitutes to her children. Mothers’ have some situation to successful and continuous breastfeeding when there is a possibility that mother may seek for alternative foods other than breastfeeding. The six most significant themes in this study were good knowledge but poor practice on breastfeeding, perceived definition and sources of information on infant formula, superficial knowledge on disadvantage of infant formula, perception of insufficient breast milk production, social and cultural aspect and hospital practice leads to formula feeding over breastfeeding.

In this study, we found that mothers appeared to have adequate knowledge about colostrum feeding, initiation of breastfeeding within 1 hour after delivery, exclusive breastfeeding up to 6 months, appropriate complementary feeding and continuation of breastfeeding till 2 years of age. Instead of having this knowledge there was clear gap exist in practice level. It was clear that there is large gaps exist in knowledge-to-practice which is not new in even Bangladeshi context. An earlier study also showed that knowledge and practice regarding proper breastfeeding is poor [35] and 90% gaps in recommended exclusive breastfeeding practices [36]. Though we did not ask any critical question to the mother probably the messages regarding the benefits of breastfeeding was not much clearer to them. A study found that infant feeding decisions have been shown to be influenced by knowledge of breastfeeding benefits [37, 38]. Nevertheless, providing proper knowledge by the health worker/health care provider along with well-planned counselling session can increase infant feeding knowledge so that mothers are solely well motivated to practice breastfeeding norms.

Participants in this study reported that they could not differentiate formula with other milk. These findings have some similarities to another study conducted by Roy *et al*., (2002) in urban slums of Dhaka city [18]. In this study, Roy and others found lower class mother are less able to differentiate powder milk and infant formula than other. Formula and tinned milk is treated as same which is frightening as a mother may feed powder milk to the infants as both are available in the container. Historically it has been seen that infant feeding practices of the rural mother are improper. For example, Mihrshahi *et*.*al* study (2010) showed that in rural Bangladesh by 2–3 months 30% of infants were giving bottles containing various kinds of milk [38]. The study identified that most participants in both groups had superficial knowledge and awareness on the harmful effect of formula feed. They only mentioned few conditions that are very common to all infants and child. Findings showed that respondents had limited knowledge of advantages and were not able to justify the reasons behind the disadvantages of infant formula. If they aware they should mention at least some major conditions like asthma, wheeze etc. that are harmful to them.

Both medical professionals and non-professionals were identified as major sources of formula feeding along with TV advertisement. However, it was not clearly identified what positive/negative messages they received from the various sources. Since mothers either hospital or home delivery have frequent contact with family members, mass media and doctors for an antenatal or postnatal checkup, enhancing their positive impact on formula feeding practices is a concern for public health intervention. However, community health worker could be an important source to enhance knowledge about infant feeding practices of mothers and her family members. Because they can have an influence on the information that is given to mothers during counselling and on the choices that they make [36]. A study revealed that women who receive information about breastfeeding from healthcare workers have a tendency to breastfeed exclusively for longer periods [39]. It is also important to educate mothers about breastfeeding before or during early pregnancy and continue after delivery [39]. This study identified one of the important findings on mother’s exposure to TV where they get information of infant formula and mothers also influenced by the photo of the healthy child on the label of formula. In another study also found the same. Commercial infant formula is treated as breast milk that baby will grow as healthy as the child on the label of the infant formula [40]. The lack of information about the benefits of breastfeeding and risks of artificial feeding, together with widespread availability and inappropriate advertising of breast milk substitutes, have a significant impact on the decisions that families make about feeding practice of children. Advertising for and marketing of breast milk substitutes can undermine a mother’s choice to breastfeed [41]. Hence, mothers require a good counsellor at all contact points that in any situation they would be promoted for the optimum duration of breastfeeding and increase the prevalence of exclusive breastfeeding [42].

Perceived insufficient breastmilk production was one of the important reasons cited by both breastfeeder and formula feeder mother in this study. Mothers own psychological issue such as a feeling of insufficient breast milk, incomplete breastfeeding has been widely recognized as the most common barrier to breastfeeding [18, 43–44] and at the same time most common reason to initiate formula before the age of 6 months. This issue is reinforced in the present study. A study specifically on this issue highlighted that mothers perception is often unwarranted if they have not any complications like postpartum haemorrhage, breast reduction surgery etc [45]. Even a mother can produce enough breast milk for single or twin baby if baby suckles the breast milk effectively as much as needed [46]. In the present study, health care providers also raised the issue quite differently with mother’s lack of confidence and improper positioning during breastfeeding. A low milk supply can result from incorrect breastfeeding techniques [45]. However, it was not identified in this study whether doctors/nurses provided any advice or medication to mothers to increasing breastmilk supply. But it can be assumed mothers did not get any advice from them. Family members and health providers should learn appropriate methods of breastfeeding and encourage mothers to continue breastfeeding. It is also very much important to further in-depth research on ‘cultural construction of insufficient breast milk production’. So that appropriate management can be undertaken to overcome this situation. In our findings, it has been showed that health care providers have some impression that mothers are somewhat impatient regarding breastfeeding her child. But it has not been triangulated in this study. However, this issue demands further exploration.

One of the major findings of this study highlighted that formula feeding has been treated as higher social status in the community, which supports a similar observation in both urban and rural Bangladesh [18, 47]. Thomas et al also assume that women who are wealthier may have an increased capability to buy infant formula [48]. When mothers believe that since rich people feed formula, it is not harmful to the health of the child, then it is difficult to motivate women and other female family members about the benefits of breastfeeding as well as demerits of formula feeding. These mothers need continuous support during the antenatal period to avoid these type of social stigma and social belief that are culturally constructed.

The attitude of family members about formula feeding plays a role in the decision of feeding method is documented in this study which is more common in another study too [18]. This type of statement or explanation is very common in low-income mothers than higher income mothers [49]. Mothers motivated on formula by the close family members as because she observed the community practice regarding infant feeding. Amir said that these unconscious experience and memories may play a powerful determinant. And these unconscious memories further drive them to behave like other community women and try to explain it by hunger of child [46]. As attitudes can determine behaviour, it is very much alarming if mother and her family members display a positive attitude towards formula feeding. It is important that the mother themselves and family members are included during health education about breastfeeding. However educational interventions are not always improved feeding practices of the mother [43].

Many studies all over the world showed that health providers especially nurses, counsellors or some at the hospital/clinic influenced mothers decision to choose formula. It is also been documented in other studies that most of the mothers received counselling from health professionals about infant feeding methods and these professionals had an influence on their infant feeding decision [15, 44]. Other hospital support characteristics, such as breastfeeding support from hospital delivery nurses, lactation specialist or peer counsellor, or receipt of free formula packets in the hospital, have also been described as important influences on women’s breastfeeding decisions [50–51]. Women who indicated that the hospital staff did not teach them how to breastfeed had more than two times greater odds of citing individual reasons than women who indicated that the staff taught them how to breastfeed[52].

In the present study, all of the formula feeder mothers had C-section and hospital delivery and got advice from the doctors to feed formula to their babies. C-section delivery legalized formula feeding in the society. Research also found an association between exclusive breastfeeding and having a cesarean delivery [44, 53]. After surgery (C-section) mothers feel pain around the incision area and there is difficulty in the movement because of catheterization and intravenous lines [54]. This could be a reason behind this association. Hospital support for early initiation and exclusive breastfeeding is tremendously important. Few studies revealed that women who receive encouragement to breastfeed from health care providers are more likely to initiate and maintain breastfeeding than women who did not receive encouragement [55]. An earlier study in Bangladesh suggested that unless the healthcare providers are prevented from suggesting BMS, it would be difficult to convince mothers for the widespread practice of breastfeeding [37]. Health providers from healthcare facilities should encourage the mother and her family to breastfeed. As Bangladesh has a shortage of health care provider in rural areas where village doctor and community health workers predominant the care services [56], it can be easily assuming that lactation consultant; paediatricians are less available for rural women.

### Study strength and limitations

The main strength of this study is that we have conducted this study in the most rural parts of Bangladesh as a baseline survey on the reason behind formula feeding by mothers. However, we cannot say the findings of this study can be similar to other rural parts of Bangladesh. This study has other limitations too. Despite the plan for FGD with the mother who were formula feeding could not interview because the appointments were not taken with them. Thus the study focused on a small number of formula feeders for the IDIs. For this reason, there is a possibility to miss other factors important to them who were more dependent on infant formula. However, we assume that the getting ‘rich data’ could be more important rather than the size of the sample. However, this was also helpful to get triangulated results which emerged from various participants through using of multiple methods. Another limitation was that this research limited its focus to formal health care providers. Later we assumed traditional healthcare providers might contribute a good number of information regarding cultural factors of infant feeding behaviour and practices. This study did not allow contextual analysis when using purposive sampling in selecting study area. However, triangulation of information was a useful approach to definite the factors on formula feeding practices of the rural mother of Bangladesh.

## Conclusions

This study provides a clear understanding of the reasons behind rural women’s initiation to formula feeding rather than breastfeeding. This study provides strong evidence which will contribute to policy making and develop more specific interventions targeting expected mother and their family members that can contribute to stop formula feeding and increase breastfeeding practices in rural Bangladesh. The study identified a wide range of factors including misunderstanding between infant formula and other powder milk, lack of appropriate breastfeeding practices, superficial knowledge on harmful effect on infant formula; the influence of family and society and authoritarian power of hospital staff. The study findings suggest that programmes can consider ensuring access to information, education and communication activities, particularly in rural areas. Publicity on the disadvantage of infant formula, benefits of breastfeeding, a misconception on insufficient milk production through electronic media, community-based popular media like street theatre; folk song may be a good option to promote breastfeeding practices. This study also highlights the importance of motivating power of family members over mother’s decision making process on infant feeding practices. This group of people should be targeted for community mobilization programme to enhance the knowledge on breast and formula feeding. Very importantly our study findings highlighted that hospital staffs, especially doctors and nurses, encourage the use of infant formula in various ways. Thus it is entirely vital to advocate and monitor their activities regularly. At the same time promotion of breast milk substitute in healthcare settings should be banned so that they cannot refer the mother to feed formula and mother and their family members cannot confuse regarding decision making on proper infant feeding practices.

Counselling on the disadvantage of unnecessary feeding of infant formula should be in accordance with IYCF awareness programme. In that case behaviour change messages should be appropriately selected taking into consideration of literacy level of the audience. As findings suggest that hospital and health care providers are not supportive of breastfeeding so emphasize should be given on enacting, enforcement and monitoring of legislation related to the code of marketing of breast milk substitute. Also, they should be trained enough on legislation and enforcement related to the inappropriate marketing of breast-milk substitute.

## Supporting information

S1 Table FGD guide for the mothers who are breastfeeding. (DOC)

S2 Table IDI guide for the mothers who are formula feeding. (DOCX)

S3 Table KII guide for health care provider. (DOCX)

S4 Table Sources of information on infant formula. (DOCX)

## References

[1] Sanjeev D, Anuradha D. Women literacy and infant feeding practices in rural integrated child development scheme (ICDS) block of Delhi. National Journal Community Medicine. 2012; 3(3):385–390.

[2] Ramachandran P. Breastfeeding practices in South Asia. The Indian Journal of Medical Research. 2004; 119(6): xiii–xv. 15243170

[3] World Health Organization. WHO Collaborative study team on the role of breastfeeding on the prevention of infant mortality effect of breastfeeding on infant and child mortality due to infectious diseases in less developed countries: A pooled analysis’. Lancet. 2000;355(9202):451–455. 10841125

[4] World Health Organization. Breastfeeding-early initiation: World Health Organization. 2012 Available from http://www.who.int/elena/titles/early_breastfeeding/en/.

[5] Oddy W, Li J, Robinson M, Whitehouse A. The long-term effects of breastfeeding on Development, Contemporary Pediatrics, Dr. Oner Ozdemir(ED.) ISBBN:978-953-51-0154-3, InTech,2012. Available from http://cdn.intechopen.com/pdfs/31651/InTech-The_long_term_effects_of_breastfeeding_on_development.pdf

[6] World Health Organization. Infant and young Child feeding. 2018. Available from http://www.who.int/news-room/fact-sheets/detail/infant-and-young-child-feeding

[7] Mullany L, Katz J, Li Y, Khatry S, Leclerq S, Darmstadt G, et al Breastfeeding patterns, time to initiation, and mortality risk among newborns in southern Nepal. Journal of Nutrition. 2008;138 (3):599–603. 10.1093/jn/138.3.599 . 18287373 PMC2366167

[8] Victora CG, Bahl R, Barros AJD, Franca GVA, Horton S, Krasevec J. Breastfeeding in the 21^st^ century: Epidemiology, mechanisms, and lifelong effect. Lancet. 2016; 387(10017):475–90. 10.1016/S0140-6736(15)01024-7 26869575

[9] Edmond KM, Zandoh C, Quigley MA, Amenga-Etego S, Owusu-Agyei S, Kirkwood BR. Delayed breastfeeding initiation increases risk of neonatal mortality. Pediatrics. 2006; 117(3):e380–e386. 10.1542/peds.2005-1496 16510618

[10] Tawiah-Agyemang C, Kirkwood BR, Edmond K, Bazzano A, Hill Z. Early initiation of breast-feeding in Ghana: barriers and facilitators. Journal of Perinatology. 2008; Supplementary 2:S46–52. 10.1038/jp.2008.173 19057568

[11] Begum M. Bresatfeeding versus formula feeding and Diarrheal diseases in infants and children-a review. Journal of Bangladesh College of Physicians and surgeons. 2014;32:26–30. 10.3329/jbcps.v32i1.21033

[12] Cai XWT, Brown DW. Global trends in exclusive breastfeeding. International Breastfeeding Journal. 2012;7:12 10.1186/1746-4358-7-12 23020813 PMC3512504

[13] Zhang K, Tang L, Wang H, Qiu L-Q, Binns CW, Lee AH. Why Do Mothers of Young Infants Choose to Formula Feed in China? Perceptions of Mothers and Hospital Staff. International Journal of Environmental Research and Public Health. 2015;12(5):4520–4532. 10.3390/ijerph120504520 . 25918908 PMC4454923

[14] Bonia K, Twells L, Halfyard B, Ludlow V, Newhook LA, Murphy-Goodridge J. A qualitative study exploring factors associated with mothers’ decisions to formula-feed their infants in Newfoundland and Labrador, Canada. BMC Public Health. 2013;13(1):645. 23844590 10.1186/1471-2458-13-645 PMC3729536

[15] Ogbuanu CA, Probst J, Laditka SB, Liu J, Baek J, Glover S. Reasons why women do not initiate breastfeeding: A Southeastern State Study. Women's Health Issues. 2009;19(4):268–278. 10.1016/j.whi.2009.03.005 . 19589476 PMC2865685

[16] Kong SKF, Lee DTE. Factors influencing decision to breastfeed. Journal of Advance Nursing. 2004;46(4):369–379. 10.1111/j.1365-2648.2004.03003.x . 15117348

[17] Shortt E, McGorrian C, Kelleher C. A Qualitative study of infant feeding decisions among low-income women in the Republic of Ireland. Midwifery. 2013;29(5):453–460. 10.1016/j.midw.2012.03.001 23142432

[18] Roy SK, Groot SD, Shafique S, Afroz A. Perceptions of mothers and use of breast milk substitutes in Dhaka, Bangladesh. Journal of Health Population and Nutrition. 2002;20(3):264–270. 12430764

[19] National Institute of Population Research and Training (NIPORT), Mitra and Associates, and ICF International.2015 Bangladesh Demographic and Health Survey 2014: Key Indicators. Dhaka, Bangladesh and Rockville, Maryland, USA: NIPORT, Mitra and Associates, and ICF International;

[20] Hossain M. Introduction in Hosain M, Kairy SN, Bayes A (edited). Driving Development A story of BRAC’s Evolution and Effectiveness. University Press Limited, 2016.

[21] Akter F and Rahman A. Role of stakeholders in promoting breastfeeding in the light of the breast milk substitute law-2013 in rural areas of Bangladesh. Available from http://research.brac.net/new/staff/role-of-stakeholders-in-promoting-breastfeeding-in-the-light-of-the-breast-milk-substitutes-bms-law-2013-in-rural-areas-of-bangladesh

[22] Banglapedia. National Encyclopedia of Bangladesh.www.banglapedia.org

[23] Income, expenditure and poverty, Bangladesh Bureau of Statistics, Statistics and Informatics Division, Ministry of Planning, Zila level poverty map estimates, 2010. Available from http://203.112.218.65/WebTestApplication/userfiles/Image/LatestReports/Bangladesh_ZilaUpazila_pov_est_2010.pdf.

[24] Bangladesh Bureau of Statistics (BBS) and UNICEF Bangladesh. Bangladesh Multiple Indicator Cluster Survey (MICS) 2012–2013, Progotir Pathey: Final Report. Bangladesh Bureau of Statistics (BBS) and UNICEF Bangladesh, 2014, Dhaka, Bangladesh.

[25] Millennium Development Goals. Bangladesh Progress Report 2015. General Economics Division (GED) Government of the Peoples Republic of Bangladesh. September 2015. Available from http://www.plancomm.gov.bd/wp-content/uploads/2015/09/MDGs-Bangladeh-Progress-Report_-PDF_Final_September-2015.pdf

[26] Ahmed A. Provision of primary health care in Bangladesh: an situational analysis. Paper presented at the conference on Development research at Lund University, 26 September 2003, Lund: Lund University: 2003.p19. Available from http://project.nek.lu.se/publications/workpap/Papers/WP03_18.pdf

[27] World Health Organization. International Code of Marketing of Breast-milk Substitutes, World Health Organization, 1981 Available from http://www.who.int/nutrition/publications/code_english.pdf.

[28] Atchan M, Foureur M, Davis D. The decision not to initiate breastfeeding—women's reasons, attitudes and influencing factors—a review of the literature. Breastfeeding Review. 2011;19(2):9–17. 22053499

[29] O’Reilly M. Parker N. Unsatisfactory saturation: A critical exploration of the notion of saturated sample sizes in qualitative research. Qualitative Research Journal. 2012;1–8. 10.1177/1468794112446106

[30] Walker JL. The use of saturation in qualitative research. Canadian Journal of Cardiovascular Nursing. 2012; 22(2), 37–46. . 22803288

[31] Guest G, Bunce A, Johnson L. How many interviews are enough? An experiment with data saturation and variability. Field Methods. 2006; 18(1), 59–82. 10.1177/1525822X05279903

[32] Rabiee F. Focus-group interview and data analysis. Proceedings of the Nutrition Society. 2004;63(4):655-660.PMID:15831139. 10.1079/pns2004399 15831139

[33] Graneheim UH, Lundman B. Qualitative content analysis in nursing research: concepts, procedures and measures to achieve trustworthiness. Nurse Education Today. 2004;24(2):105–112. 10.1016/j.nedt.2003.10.001 . 14769454

[34] Shenton AK. Strategies for ensuring trustworthiness in qualitative research projects. Education for information. 2004; 22:63–75. Available from https://pdfs.semanticscholar.org/452e/3393e3ecc34f913e8c49d8faf19b9f89b75d.pdf

[35] Haider R, Rasheed S, Sanghvi TG, Hassan N, Pachon H, Islam S, et al Breastfeeding in infancy: identifying the program-relevant issues in Bangladesh. International Breastfeeding Journal. 2010;5:21 10.1186/1746-4358-5-21 . 21118488 PMC3009955

[36] Ahmed S, Islam A, Parveen SD. Infant feeding practices in rural Bangladesh: policy implications. Working Paper 146. 1998, ICDDRB. Available from http://pdf.usaid.gov/pdf_docs/pnacm636.pdf. 10.1093/tropej/45.1.37 10191591

[37] Street DJ, Lewallen LP. The influence of culture on breast feeding decisions by African American women and white women. Journal of Perinatal and Neonatal Nursing. 2013;27(1):43–51. 10.1097/JPN.0b013e31827e57e7 . 23360941

[38] Mihrshahi S, Kabir I, Roy SK, Agho KE, Senarath U, Dibley MJ, et al Determinants of infant and young child feeding practices in Bangladesh: Secondary data analysis of demographic and health survey 2004. Food & Nutrition Bulletin. 2010; 31(2):295–313. 10.1177/156482651003100220 . 20707235

[39] Ludvigsson JF. Breastfeeding in Bolivia–information and attitudes. BMC Pediatrics. 2003;3(4):1−12. 10.1186/1471-2431-3-4 . 12769829 PMC161813

[40] Prak S, Dahl MI, Oeurn S, Conkle J. Wise A, Laillou A. Breastfeeding Trends in Cambodia, and the Increased Use of Breast-Milk Substitute—Why Is It a Danger? Nutrients.2014;6(7):2920–2930. 10.3390/nu6072920 . 25054552 PMC4113769

[41] Adhikari T, Subedi I. Knowledge and practice on breastfeeding among mothers of infant. Journal of Food Science and Technology Nepal. 2013;8:71–74. 10.3126/jfstn.v8i0.11754

[42] Amir LH. Breastfeeding-Managing “supply” difficulties. Australian family physician. 2006;35(9):686–689. . 16969436

[43] Bennington LK. Breastfeeding multiples: it can be done. Newborn and Infant Nursing Reviews. 2011;11:1947.

[44] Hackett KM, Mukta US, Jalal CSB, Sellen DW. A qualitative study exploring perceived barriers to infant feeding and care giving among adolescent girls and young women in rural Bangladesh. BMC Public Health. 2015;15:771 10.1186/s12889-015-2115-5 26259575 PMC4531479

[45] Li R, Fein SB, Chen J, Grummer-Strawn LM. Why mothers stop breastfeeding: mothers’ self-reported reasons for stopping during the first year. Pediatrics. 2008; 122(Suppl 2):S69–76. 10.1542/peds.2008-1315i . 18829834

[46] Amir LH. Social thory and infant feeding. International breastfeeding Journal. 2011; 6:7 10.1186/1746-4358-6-7 21676218 PMC3125336

[47] Chezem J, Friesen C, Clark H. Sources of infant feeding information used by pregnant women’. Journal of Perinatal Education. 2001;10(3):20−26. 10.1624/105812401X88291 17273262 PMC1595081

[48] Thomas JS, Yo EA, Tirmizi N, Owais A, Das SK, Rahman S, et al Maternal knowledge, attitude and self-efficacy in relation to intention to exclusively breastfeed among pregnant women in rural Bangladesh. Maternal and Child Health Journal. 2015; 19 (1): 49–57. 10.1007/s10995-014-1494-z 24752315

[49] Khoury AJ, Moazzem SW, Jarjoura CM, Carothers C, Hinton A. Breast-feeding initiation in low-income women: Role of attitudes, support, and perceived control. Womens Health Issues. 2005;15(2):64–72. 10.1016/j.whi.2004.09.003 15767196

[50] Joshi PC, Angdembe MR, Das SK, Ahmed S, Faruque ASJ, Ahmed T. Prevalence of exclusive breastfeeding and associated factors among mothers in rural Bangladesh: a cross-sectional study. International Breastfeeding Journal.2014;9:7 10.1186/1746-4358-9-7 24904683 PMC4046052

[51] Qiu L, Zhao Y, Binns CW, Lee AH, Xie X: Initiation of breastfeeding and prevalence of exclusive breastfeeding at hospital discharge in urban, suburban and rural areas of Zhejiang China. International Breastfeeding Journal. 2009;4:1 10.1186/1746-4358-4-1 19175909 PMC2637253

[52] Shinwell ES, Churgin Y, Shlomo M, Shani M, Flidel-Rimon O: The effect of training nursery staff in breastfeeding guidance on the duration of breastfeeding in healthy term infants. Breastfeeding Medicine. 2006;1(4):247–52. 10.1089/bfm.2006.1.247 17661605

[53] Ahmed SM, Hossain MA, Chowdhury AMR, Bhuiya AD. The health workforce crisis in Bangladesh: shortage, inappropriate skill-mix and inequitable distribution. Human Resources for Health. 2011;9:3 10.1186/1478-4491-9-3 21255446 PMC3037300

[54] Save the Children: Breast-milk substitutes Threaten young lives. Available from: https://sierraleone.savethechildren.net/sites/sierraleone.savethechildren.net/files/library/SL%20BREASTFEEDING%20Report.pdf.

[55] Foss KA, Southwell BG. Infant feeding and the media: the relationship between Parents' Magazine content and breastfeeding, 1972–2000. International Breastfeeding Journal. 2006; 30:1:10. 10.1186/1746-4358-1-10 PMC1489921 16722542

[56] Susiloretni KA, Krisnamurni S, Sunarto, Widiyanto SY, Yazid A, Wilopo SA. The Effectiveness of Multilevel Promotion of Exclusive Breastfeeding in Rural Indonesia. American Journal of Health Promotion. 2013;28(2):e44–55. 10.4278/ajhp.120425-QUAN-221 23621652

