# Supplementary material for: Reasons for formula feeding among rural Bangladeshi mothers: A qualitative exploration
Source: PLoS One. 2019 Feb 26;14(2):e0211761. doi: 10.1371/journal.pone.0211761 (PMC6391007; doi:10.1371/journal.pone.0211761)
Supplement: S1 Table — (DOC) [file pone.0211761.s001.doc]

**S1 Table:** FGD guide for mother

| Introduction | Welcome  Introductory information about the study  Consent |
| --- | --- |
| Personal history of the participants | Age, gender, educational qualification, current occupation, |
| Major questions | - What do you know about breast feeding (Probe: initiation of breast feeding, colostrum feeding and its importance, pre-lacteal feeding, milk exclusive breast feeding, duration of breast feeding, sources of breastfeeding, advantage and disadvantage of breastfeeding) - How are you feeding your baby? (Probe: breastfeeding exclusively, both breastfeeding and breast-milk substitutes, feeding breast-milk substitute only) - Has your baby been given anything other than breast milk since it was born? If yes, why was your baby given the supplements? How they fed? (bottle, spoon). If no why? - Have you faced any problem or what other woman/ mother faced problem regarding breastfeeding? If yes please describe (probe: personal, social, familial, financial, cultural, institutional etc.) - Why and how did you/a mother (in your community) initiate formula feeding? How they influenced? From which sources the mother heard about infant formula? - Do you have any suggestions regarding promoting breastfeeding practices and/or discourage formula feeding? Please explain. |
| Closing questions | - Do you feel there is something important we should have asked that we did not address? - Thanks |

**2: Knowledge and practice on formula feeding**

- What do you know about formula feeding? (Probe: when to feed, how they feed, how they know about formula feeding)
- Please ask some advantages and disadvantages of formula feeding (**PLEASE do not push participants to answer**)

**3: Barriers in breastfeeding practices**

- Did/do you face any problem during breastfeeding/continuing breastfeeding? (Probe: How do they manage when face any difficulties)

**4:** **Reasons behind formula feeding/Facilitating factors of formula feeding**

- What is your perception and observation why some mothers provide infant formula to their children?
- Do you have any personal experience regarding infant formula? If yes please share.

**5: Closing Questions**

- Do you have any recommendation regarding breast feeding/formula feeding/or issues related to feeding practices?
- Do you feel there is something important we should have asked that we did not address?

***[Thank you very much for your time]***
