# Supplementary material for: Reasons for formula feeding among rural Bangladeshi mothers: A qualitative exploration
Source: PLoS One. 2019 Feb 26;14(2):e0211761. doi: 10.1371/journal.pone.0211761 (PMC6391007; doi:10.1371/journal.pone.0211761)
Supplement: S2 Table — (DOCX) [file pone.0211761.s002.docx]

**S2 Table: Interview schedule for formula feeder mother**

| Introduction | Welcome  Introductory information about the study  Consent |
| --- | --- |
| Personal history of the participants | Age, gender, educational qualification, current occupation, |
| Major questions | - What do you know about breast feeding (Probe: initiation of breast feeding, colostrum feeding and its importance, pre-lacteal feeding, milk exclusive breast feeding, duration of breast feeding, sources of breastfeeding, advantage and disadvantage of breastfeeding) - How are you feeding your baby? (Probe: breastfeeding exclusively, both breastfeeding and breast-milk substitutes, feeding breast-milk substitute only) - Has your baby been given anything other than breast milk since it was born? - If yes, why was your baby given the supplements? How they fed? (bottle, spoon) - Have you faced any problem or what other woman/ mother faced problem regarding breastfeeding? If yes please describe (probe: personal, social, familial, financial, cultural, institutional etc.) - Why and how did you/a mother (in your community) initiate formula feeding? How they influenced? From which sources the mother heard about infant formula? - Do you have any suggestions regarding promoting breastfeeding practices and/or discourage formula feeding? Please explain. |
| Closing questions | - Do you feel there is something important we should have asked that we did not address? - Thanks |
