# Supplementary material for: Reasons for formula feeding among rural Bangladeshi mothers: A qualitative exploration
Source: PLoS One. 2019 Feb 26;14(2):e0211761. doi: 10.1371/journal.pone.0211761 (PMC6391007; doi:10.1371/journal.pone.0211761)
Supplement: S3 Table — (DOCX) [file pone.0211761.s003.docx]

**S3 Table: Interview schedule for Health care provider**

| Introduction | Welcome  Introductory information about the study  Consent |
| --- | --- |
| Personal history of the participants | Age, gender, educational qualification, current occupation, |
| Major questions | - Do you think mother and caregiver of the children in your area have enough knowledge on breastfeeding practices? Please explain with some examples. - What are the practices of the mother (both hospital delivery and home delivery; normal or C-section) regarding breastfeeding (Probe: initiation of breast feeding, baby been given anything other than breast since born, milk exclusive breast feeding, duration of breast feeding) - What are the practices of the mother (both hospital delivery and home delivery; normal or C-section) regarding formula feeding (Probe: initiation of breast feeding, baby been given anything other than breast since born, exclusive breast feeding, duration of breast feeding) - Do any mothers/caregivers seek suggestion about child feeding practices to you? - What type of problem they face? Or what type of suggestion they seek for? (Probe: Example as much as possible) - How do you encourage women regarding breastfeeding (Probe: mother who have delivered at hospital, mother who take consultation for their infants and child, caregiver/fathers) - What do you think what is the influencing factors of infant feeding practices? (Probe: social, cultural, financial, institutional, media etc.) - Why and how did a mother (in this community) initiate formula feeding? How they influenced? From which sources the mother heard about infant formula? - Do you have any suggestions regarding promoting breastfeeding practices and/or discourage formula feeding? Please explain. |
| Closing questions | - Do you feel there is something important we should have asked that we did not address? - Thanks |
