# Supplementary material for: Reasons for formula feeding among rural Bangladeshi mothers: A qualitative exploration
Source: PLoS One. 2019 Feb 26;14(2):e0211761. doi: 10.1371/journal.pone.0211761 (PMC6391007; doi:10.1371/journal.pone.0211761)
Supplement: S4 Table — (DOCX) [file pone.0211761.s004.docx]

**S4 Table: Sources of information on infant formula**

| **Theme: Sources of information on infant formula** | |  |
| --- | --- | --- |
| **Respondent ID** | **Source of Information on infant formula** | **Selected quotes** |
| KII_DSF_1 | Ad in radio, TV, Doctor |  |
| KII_DSF_2 | Aya, ward boy, advertisement in TV, relative |  |
| KII_DSB_3 | TV add | Mother are used to get some idea from TV about ‘N’, and other branded formula |
| KII_DSB_4 | TV add |  |
| KII_DJS_5 | One to other mothers, relatives | When baby start to cry after birth and instantly not get mother milk then family members and some of their relatives told them to feed formula and they think it is not harmfull for baby because they already fed their baby |
| KII_DJS_6 | TV add |  |
| KII_DJS_7 | Doctor |  |
| KII_DJS_8 | TV add, nurses, other than doctor, Private clinic or hospital |  |
| KII_NSF_9 | Mother to mother, doctor, drug seller/shopkeeper, TV add |  |
| KII_NSB_10 | TV add, drug seller in pharmacy |  |
| KII_NJS_11 | Drug seller, TV add, village doctor, relatives and neighbor |  |
| KII_NJS_12 | Village doctor, drug seller in pharmacy, advertisement in TV, family members who already used BMS, TBA |  |
| MFF-1 | Doctor, elder sister | All children of my brothers and sisters are formula feed. My brother also bought formula from market for me. |
| MFF-2 | Doctor, TV add |  |
| MFF_3 | Doctor, TV add |  |
| MFF_4 | Nurses |  |
| FGD_MSF_1 | Doctors suggestion, TV add, mother to mother |  |
| FGD_MSB_2 | TV add, relatives, doctor |  |
| FGD_MJS_3 | Relatives, TV add, Doctor, mother to mother |  |
| FGD_MJS_4 | TV add, mother to mother |  |
| FGD_FSF_1 | Doctor, Relatives, mother to mother |  |
| FGD_FSB_2 | TV add, doctor ,relatives |  |
| FGD_FJS_3 | Attractive TV add, doctor |  |
| FGD_FJS_4 | TV add, Mother to mother, doctors' suggestion and shopkeeper influence |  |
| FGD_CSF_1 | Doctors' suggestion |  |
| FGD_CSB_2 | Doctors' suggestion | In case of sickness, doctor suggest to feed formula. I believe infant formula can be given to the baby only if it is suggested by doctors |
| FGD_CJS_3 | Mother to mother, TV add, Doctor | In case of formula feeding I think influence of doctors suggestion is mostly common and then another respondent said that No mothers willingness is most prominent. |
| FGD_CJS_4 | Doctors' suggestion | When my baby was sick (after few days of delivery) I went to doctor and he suggested me to feed formula |
